# Innovation and authenticity: Constructing tourists’ subjective well-being in festival tourism

**DOI:** 10.3389/fpsyg.2022.950024

**Published:** 2022-09-20

**Authors:** Shu-Ning Zhang, Fang Deng

**Affiliations:** College of Tourism, Huaqiao University, Quanzhou, China

**Keywords:** festival tourism, subjective well-being, innovation, festival authenticity, experience

## Abstract

Although festival tourism is an excellent fertile ground for improving individual emotions, few studies have been conducted on the influencing factors and formation mechanisms of festival tourists’ subjective well-being. To address the current research gap, this paper draws on Arnold’ s theory of emotion to examine a comprehensive formation model of tourists’ subjective well-being. The findings from 581 samples indicate that event design innovation, cultural innovation and aesthetic innovation of festival tourism are positive stimulus factors of tourists’ subjective well-being. Both experience quality and perceived festival value mediate the effects of cultural innovation and aesthetic innovation on subjective well-being, yet have no mediating effect on the relationship between event design innovation and well-being. However, it can only be achieved when festival authenticity contributes to a positive moderating effect. This study provides new ideas for the collaborative advancement of innovative development and authentic inheritance in festival tourism destinations.

## Introduction

Festival tourism refers to tourists participating in leisure and festive activities during festivals (Kruger and Viljoen, 2021). This kind of activity is usually held only once or several times a year and lasts for a limited time, providing attendees with unusual networking opportunities (O’Sullivan and Jackson, 2002). Festival tourism activities are characterized by elements such as festival culture, unique experience atmosphere, entertainment space that attract many tourists to experience the celebration (Lin and Lee, 2020). Attending festivals can not only release stress, satisfy spiritual enjoyment and create rare social opportunities, but also provide positive experiences such as accumulating festival knowledge, satisfying aesthetics and curiosity (Quinn, 2006; Li et al., 2020; Choi et al., 2021). Previous study has shown that subjective well-being is significantly positively correlated with activities that enhance social interaction and interpersonal relationships, as well as individual positive experience (Yolal et al., 2016). Obviously, festival experience is an excellent channel for cultivating tourists’ subjective well-being, which ultimately affects the long-term successful operation of festival tourism. However, there are few studies have focused on tourists’ subjective well-being in festival tourism.

Research to date has majorly discussed the effects of festival tourism, festival management, sustainable development, consumer behavior, etc. (Quinn, 2006; Tanford and Jung, 2017). Especially from a micro perspective, the tourist experience and emotional intentions are hot topics in festival tourism research. Many scholars are concerned with festival tourism motivation, tourist experience elements, uniqueness perception, attachment level (Lau and Li, 2019), tourist satisfaction, loyalty and revisit intentions after the experience (Girish and Chen, 2017). However, as the positive effect of the tourist experience, happiness can measure the emotional and spiritual experience beyond pleasure (Saragih and Amelia, 2020). It not only marks tourists’ affirmation of the meaning and value of an experience but also indicates their special emotion toward travel destinations (Wu et al., 2017). Accordingly, tourists’ subjective well-being, as a deep-level travel emotional response, represents a high level of the overall experience quality and quality of life (Yolal et al., 2016; Anglim et al., 2020; Kwon and Lee, 2020). This can spark tourists’ good travel memories and bring extra surprises (Kim et al., 2020; Kwon and Lee, 2020), which is conducive to word-of-mouth communication and revisit intention. However, research focuses on positive antecedents of festival tourists’ subjective well-being is still limited (Laing, 2018). Therefore, this study aims to fill the current research gap by exploring the positive influencing factors of tourists’ subjective well-being.

Richards and Wilson (2006) argue that many tourists are tired and repulsed by tourist destinations using formulaic methods to shape and promote local cultural characteristics. The reason is that the duplication of tourism culture and product homogeneity will greatly affect their impressions and experiences at a destination. Therefore, scholars have called on tourist destinations to adopt innovative methods to avoid these negative situations (Stamboulis and Skayannis, 2003; Richards and Wilson, 2006). Meanwhile, many festival tourists seek a fresh and exciting festival experience (Trinh and Ryan, 2016). Innovation is in line with the direction of future demand and essential to tourists’ spiritual enjoyment (Gardiner and Scott, 2018). Moreover, the positive correlation between innovation attributes and tourists’ subjective well-being has been verified (Kim et al., 2020). Similarly, innovations in festival tourism, such as event design innovation and the integration of cultural and creative elements, can cater to the experiential motivation, providing tourists with excitement and pleasure (Stamboulis and Skayannis, 2003; Hjalager, 2010; Fu et al., 2018). They may even inspire a sense of well-being in tourists. Hence, this paper will examine the influence path and process mechanism of festival tourism innovation on tourists’ subjective well-being.

The current study aims to address the following issues to fill the research gap: (1) Can festival tourism innovation affect tourists’ subjective well-being? (2) What kind of mediating constructs exist between innovation and subjective well-being? (3) What factor plays a critical moderating role in the festival tourism experience? In this way, this study introduces three independent variables (i.e., event design innovation, cultural innovation, and aesthetic innovation) to represent festival tourism innovation, and uses experience quality and perceived festival value as the dual mediating variables. Furthermore, festival authenticity is used to be the moderating variable to construct a theoretical model of the influence of festival tourism innovation on tourists’ subjective well-being. Theoretically, from an innovation perspective, this research explores the formation mechanism of tourists’ subjective well-being in the context of festival tourism and clarifies the process mechanism and internal logic between innovation and tourists’ subjective well-being. Practically, we provide theoretical guidance for improving the positive emotions and happiness of festival tourists. The findings show significant implications for enhancing the competitiveness of the festival tourism market and provide new ideas for the collaborative advancement of innovative development and authentic inheritance in festival tourism destinations.

## Literature review and hypothesis

### Festival tourism innovation

Innovation is understood as generating, accepting and implementing new ideas, as well as resulting in new products, services or transactions, etc. (Hjalager, 2010). Unsurprisingly, for organizations, innovation has long been the best mechanism for coping with fierce competition and an effective strategy for achieving sustainable growth (Schumpeter, 1934). Hjalager (2010) summarizes five levels of innovation, including product/service innovations, process innovations, managerial innovations, management innovations and institutional innovations. However, given that the core business provided by tourism is often intangible, non-preservable and easily replaceable, most of the classic innovation literature focuses on how to provide attractive product and service concepts (Hjalager, 2010; Yang and Tan, 2017). “Seeking novelty” has long been one of the key driving forces for most tourists’ travel (Assaker et al., 2011). Scholars propose that “creating new experiences for tourists” is a critical direction in the future and discuss this topic in the context of virtual tourism and cultural and creative tourism (Lee, 2010; Yang and Tan, 2017; Kim et al., 2020; Zhang et al., 2021). Therefore, relevant conclusions have strengthened the value of tourism activity innovation at the tourist experience level, such as improving emotional value, functional value, learning desire, host-guest interaction, satisfaction, etc. (Yang and Tan, 2017; Yeh et al., 2019; Kim et al., 2020; Zhang et al., 2021). Festival tourism is a regular and grand festive event that brings together a group of attendees with similar experience goals and provides them with an atmosphere for an unforgettable experience (Kruger and Viljoen, 2021). Festival tourism innovation not only satisfy tourists’ new experience and creates destination revenue, but also helps the sustainable development of festivals. However, there is still a lot of room for innovative research in the context of festival tourism. Although scholars have identified the significance of innovation and are concerned about innovation in festival organization and management (Larson, 2009; Hjalager, 2010), they rarely discuss the impact of festival tourism innovation on tourists’ cognition and emotion from a micro perspective. Therefore, the purpose of this paper is to consider whether and how festival tourism innovation affects tourists’ experience evaluation. Therefore, the purpose of this paper is to examine whether and how festival tourism innovation affects the post-experience feelings of tourists.

First, Tanford and Jung (2017) assert that events are one of the most central festival attributes, accounting for most of tourists’ travel arrangements. Their fondness for projects and activities largely affects the overall travel experience quality and positive emotions (Fu et al., 2018). For example, the quality of the core activities (music performances) of traditional music festivals is one of the key criteria for forming a positive word of mouth among tourists. The principal activities of the food festival, such as cooking and tasting, also leave the deepest impression on the attendees (Hu, 2010). Event design innovation is tourists’ creative perception of performance, time arrangement, venue arrangement, etc. during festivals (Fu et al., 2018). Festivals that provide exciting and creative event design can meet the needs of tourists for innovative experience and stimulate their positive emotions. Secondly, Liu et al. (2019) has proved that tourists have a strong desire to learn about culture and customs, and has concluded that cultural experience is an essential content and core competitiveness of festival tourism. Cultural innovation is by no means blindly abandoning the festival itself, but adopting a positive attitude while inheriting the essence of festival culture and accepting other excellent cultures (Hu, 2010; Lee, 2010). Therefore, for most tourists who pursue cultural experience in festival tourism, cultural innovation is an effective channel to cultivate positive emotions (Zhang et al., 2019c). Finally, the aesthetic experience is an essential element of festival tourism, and it runs through the entire period of the tourist experience (Aşan et al., 2020). Aesthetic innovation enables tourists to feel and integrate into the festival atmosphere, implanting more comfortable imagination space for tourists (Hu, 2010). Most importantly, tourists’ aesthetic participation during festival celebrations is passive (Zhang and Xu, 2020), which leads to aesthetic innovations more likely to affect their cognition and emotion. This paper selects representative event design innovation, cultural innovation and aesthetic innovation to represent festival tourism innovation.

### Theoretical background

Arnold’s theory states that the individual brain processes stimulating information about events or phenomena to trigger positive or negative emotions (Arnold, 1960). Subjective well-being, as one of the best experiences for tourists, is also understood as the degree to which positive emotions overcome negative emotions (Saragih and Amelia, 2020). It can be seen that Arnold’s theory can provide theoretical evidence for the formation mechanism of tourists’ subjective well-being (Choi and Choi, 2019). More importantly, Arnold’s theory emphasizes that the basic link of emotion formation is “stimulus-evaluation-emotion” (Arnold, 1960; Choi and Choi, 2019), which means that subjective well-being is the result of tourists’ evaluation of stimulus information.

Hjalager (2010) argues that innovation always occurs in complex networks in order to satisfy many festival participants with various interests. Although it has to be acknowledged that innovation cannot cater to all visitors, competition between festivals and other experiential events is increasing (Hjalager, 2010; Gardiner and Scott, 2018). If the festival is not innovative, it will be more difficult to attract repeat customers, and its festival quality will be difficult to improve. Therefore, according to Arnold’s theory, this study considers festival tourism innovation as the positive stimulus condition. Under the stimulation of innovation, the cerebral cortex of tourists will perceive the elements of innovation and process information. Experience quality and perceived value are specific evaluation contents after stimulation (Chen and Chen, 2010; Aşan et al., 2020). The higher the experience quality and perceived value, the more positive emotions tourists generated, that is, the more likely they are to produce subjective well-being (Jin et al., 2015; Hussein and Hapsari, 2021). Furthermore, authenticity is another stimulus that distinguishes innovation in festival travel. Generally, exposure to authentic elements of festival culture contributes to a unique and high-quality experience for tourists (Akhoondnejad, 2016). This paper believes that festival tourism maintains a certain authenticity in the process of innovation. Therefore, authenticity, a moderator variable, plays a situational role between festival tourism innovation stimulus and “evaluation.”

**FIGURE 1:**
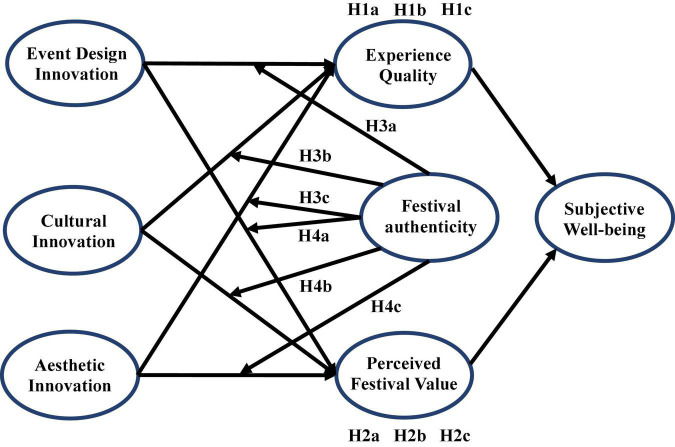
Research conceptual framework.

In summary, the formation of tourists’ subjective well-being during festival tourism involves some critical elements (i.e., innovation, experience, value, and authenticity). Specifically: (1) event design innovation, cultural innovation and aesthetic innovation form an externally stimulating environment that affects tourists’ cognition, evaluation and internal response. Tourists then evaluate the experience quality and perceived festival value after their comprehensive consideration; (2) affected by innovative stimulation, tourists’ experience and perceived festival value will be of high quality, resulting in positive emotional reactions and subjective well-being; (3) while innovating in festival tourism, maintaining authenticity can contribute to enhancing experience quality and perceived festival value. Based on this, this study constructs a theoretical model of the formation of festival tourists’ subjective well-being (as shown in Figure 1).

### Mediating role of experience quality

Experience quality is a subjective evaluation based on tourists’ overall experience in tourism activities, which has received great attention (Chen and Chen, 2010). Tourists are interested in innovative ideas because they can achieve high-quality and differentiated experiences and products (Hjalager, 2010). Festival tourism innovation can change the type of activities and create attractive products (Romão and Nijkamp, 2019). Therefore, most tourist destinations regard innovation as the key to gaining the favor of tourists and improving the quality of the tourism experience.

For instance, Larson (2009) notes that the cancelation or untimely changes to festival activities will affect experience quality, leading to reduced interest by tourists and a decrease in the number of future visitors. In contrast, enjoyable activities increase the enthusiasm and interaction opportunities of tourists and even stimulate their revisit intention (Hjalager, 2015; Romão and Nijkamp, 2019). Therefore, event design innovation is an important factor in achieving a high-quality festival tourism experience. Additionally, scholars argue that cultural innovation can help express cultural meanings and provide great convenience for the interaction between tourists and cultural elements (Hu, 2010; Gordin and Dedova, 2014). Similarly, study has shown that cultural innovation has a positive impact on tourists’ attitudes and behaviors and easily causes tourists to provide high ratings voluntarily, as destinations offer more surprises to tourists by exploring unique festival cultural elements and integrating culture, art and technology (Gordin and Dedova, 2014). Thus, cultural innovation may be another significant factor of experience quality. Similarly, aesthetic experience is a kind of “sensory science”. Tourists will have subjective sensory aesthetic experiences during festival tourism. According to Arnold’ s theory, tourists’ perception and evaluation of the experience will be better if they are stimulated by aesthetic innovation, thereby affecting their evaluation of experience quality and promoting positive behavior (Trinh and Ryan, 2016). Zhang and Xu (2020) also confirm that esthetic experience influences tourists’ attitudes and aesthetic innovation helps improve tourists’ aesthetic awareness. Thus, it can be concluded that the aesthetic innovation will positively affect the experience quality.

It is worth noting that the experience quality includes immersion, participation, surprise and fun, which are closely related to the positive psychology and behavior of tourists (Jin et al., 2015; Hussein and Hapsari, 2021). The pleasure and subjective well-being of tourists represent the best experience state (He et al., 2020). Cole and Scott (2004) point out that the positive experience of tourists has a certain cumulative effect, which can cause a leap in the emotional experience. The impact of experience quality may not stop at the level of shallow emotional responses. When positive emotions reach a certain level, tourists will have subjective well-being; that is, the experience quality may play an important role in the transmission of festival tourism innovation and tourists’ subjective well-being. Therefore, we propose the following hypotheses:

*Hypothesis 1a*: Experience quality will mediate the relationship between event design innovation and tourists’ subjective well-being.

*Hypothesis 1b*: Experience quality will mediate the relationship between cultural innovation and tourists’ subjective well-being.

*Hypothesis 1c*: Experience quality will mediate the relationship between esthetic innovation and tourists’ subjective well-being.

### Mediating role of perceived festival value

In the context of festival tourism, perceived value is the cognitive result of visitors comparing the costs and benefits after participating in festivals (Aşan et al., 2020). Innovation will create additional value for tourists and reduce the perception of non-monetary costs (Chen and Chen, 2010; Hjalager, 2010), which indicates that festival tourism innovation can enhance tourists’ perception of festival value.

More specifically, it has been found that the innovation of festival themes, festival content, and festival forms can increase the attention and involvement of tourists (Fu et al., 2018). Furthermore, creative festival performances enable tourists to have a personalized and customized festival experience, enhancing the novelty and entertainment of tourists’ activities (Hjalager, 2015; Romão and Nijkamp, 2019). Lee et al. (2011) find that festival activities have a positive impact on tourists’ functional value and emotional value. Therefore, event design innovation is likely to be an important factor in tourists’ perception of festival value. Furthermore, cultural innovation does not mean abandoning tradition but rather improving the authenticity of festival culture through innovation, which helps to make the destination more attractive to tourists (Larson, 2009; Zhang et al., 2021). While tourism cultural repetition and homogeneity have been rejected by tourists (Richards and Wilson, 2006), innovation has become a new way for tourists to acquire genuine festival cultural experiences and explore festival cultural elements. That is because the realization of activities and the presentation of festival tourism culture in an innovative way can make it easier for tourists to understand and learn about festival culture and strengthen the perception of festival value. Therefore, this study assumes that cultural innovation will benefit tourists’ perception of festival value. Additionally, some studies assert that the aesthetic perception of festival visitors comes from the visual experience (Zhang and Xu, 2020); thus, aesthetic innovation increases the aesthetic sense of visitors through aesthetic differences and novelty. The new content brought by aesthetic innovation will affect the visual experience of visitors in a pleasant way, enhancing visitors’ appreciation of the aesthetics of the festival environment and producing positive emotions (Zhang and Xu, 2020). In addition, Aşan et al. (2020) point out that the aesthetic experience is the key driver of the satisfaction and positive perceived festival value of tourists. Meanwhile, it is the aesthetic innovation of cultural products that will be recognized by and generate loyal from consumers. Therefore, this research speculates that aesthetic innovation is a necessary factor in the promotion of tourists’ perception of festival value.

In the context of festival tourism, innovation can create more possibilities between perceived festival value and tourists’ subjective well-being. Importantly, Schwartz and Sortheix (2018) have posited that perceived value and subjective well-being have a strong inner correlation because tourists will be more satisfied with their pursuit of value. Therefore, based on Arnold’s theory, after tourists are stimulated by innovation, higher festival value evaluation will stimulate positive emotions, thereby affecting tourists’ subjective well-being. We speculate that perceived festival value plays a mediating role between festival tourism innovation and the subjective well-being of tourists.

*Hypothesis 2a*: Perceived festival value will mediate the relationship between event design innovation and tourists’ subjective well-being.

*Hypothesis 2b*: Perceived festival value will mediate the relationship between cultural innovation and tourists’ subjective well-being.

*Hypothesis 2c*: Perceived festival value will mediate the relationship between aesthetic innovation and tourists’ subjective well-being.

### Moderating role of festival authenticity

Authenticity is widely discussed in the fields of psychology, sociology, and management and has also attracted much attention in tourism research (Zhang et al., 2019c; Stepchenkova and Belyaeva, 2021). Specifically, authenticity indicates the true level of products and experiences that tourists perceive. Research has shown that tourists’ choices of destinations are influenced by authenticity, and the pursuit of authenticity has become one of the tourism motives of travelers today (Stepchenkova and Belyaeva, 2021). In addition, authenticity affects the experience quality, satisfaction, and perceived value (Brida et al., 2013; Akhoondnejad, 2016). Therefore, festival authenticity has become a key factor for tourists to evaluate quality and value.

There is no doubt that event design innovation, cultural innovation and aesthetic innovation bring new experiences and excitement to tourists. They will also look forward to the uniqueness, atmosphere and originality of festivals (Akhoondnejad, 2016). It is authentic festival music, dances, costumes, performances, and crafts that can create an intriguing and unique festival cultural atmosphere. The greater the authenticity of the festival, which signifies that innovation does not deviate from the expectations of tourists, the stronger the interest of tourists in participating in experiences and interactions, and the deeper their sense of participation and real connection (Lin and Lee, 2020). Girish and Chen (2017) emphasize that the authenticity of festival activities, facilities and atmosphere have an impact on the positive emotions of tourists. The greater the festival authenticity, the more acceptance and recognition of innovative initiatives by tourists, and the more actively they will form positive emotions, cognitions and evaluations. Therefore, this study proposes the following hypotheses:

*Hypothesis 3a*: Festival authenticity moderates the positive relationship between event design innovation and experience quality.

*Hypothesis 3b*: Festival authenticity moderates the positive relationship between cultural innovation and experience quality.

*Hypothesis3c*: Festival authenticity moderates the positive relationship between aesthetic innovation and experience quality.

There is also a close correlation between authenticity and the perceived value of tourists (Akhoondnejad, 2016). Zhang et al. (2019c) emphasize that authenticity is an important influencing factor when tourists assess the value of cultural tourism destinations. Authenticity implies credibility; the higher the festival authenticity, the more tourists will trust the new content and rich travel experience with innovation. In addition, festival authenticity helps tourists accept festival tourism innovation and pushes them to learn more about local characteristics and authentic customs and rituals, appreciate festival attractions such as costumes and arts, further understand festival culture and meaning (Domínguez-Quintero et al., 2020). It ultimately enhances the positive impact of festival tourism innovation on the perception of festival value. Therefore, this study proposes the following hypotheses:

*Hypothesis 4a*: Festival authenticity moderates the positive relationship between event design innovation and perceived festival value.

*Hypothesis 4b*: Festival authenticity moderates the positive relationship between cultural innovation and perceived festival value.

*Hypothesis 4c*: Festival authenticity moderates the positive relationship between aesthetic innovation and perceived festival value.

## Research methodology

### Sample and data collection

We conducted offline surveys in two cities (Quanzhou and Xiamen) in Fujian Province, China. The major reason is that Fujian has strong regional festival characteristics and festival customs (Zhang et al., 2019c). Quanzhou and Xiamen have popular world heritage destinations, attracting a large number of cultural tourists. Festival tourism development has advantages in atmosphere and source of tourists. For example, during the Mid-Autumn Festival, these two cities have organized a unique cultural activity that lasts more than 1,300 years: moon-cake gambling. At present, the activities and cultural display forms of moon-cake gambling have become diversified and are loved by many festival tourists. For this reason, the above research areas are typical and representative.

Researchers conducted on-site surveys in tourist attractions in Xiamen and Quanzhou from September to October 2021 and majorly did the following important work. First, to obtain the real feelings of tourists, three researchers used non-probabilistic convenience sampling technique to collect questionnaires. In Quanzhou Street, Wudian Traditional Blocks, and Kulangsu Island, respondents were invited to fill out the questionnaire. Second, the researchers informed respondents that their privacy was fully protected and the answers were unbiased. Third, researchers answered any questions respondents had and stated that the answers were only used academic research to earn their trust and take them seriously. A total of 640 questionnaires were distributed and recovered (320 questionnaires were collected in Quanzhou and 320 in Xiamen), and 59 invalid questionnaires were excluded (e.g., obviously consistent answers, random selection of answers, or short answering time). Finally, 581 valid samples were obtained. The demographic characteristics of the sample in this study were shown in Table 1.

**TABLE 1:** Background of participants.

Items	Frequency	Percent	Items	Frequency	Percent
**Gender**			**Income/per month (¥)**		
Male	240	41.3%	3000 or below	251	43.2%
Female	341	58.7%	3001 ∼ 5000	132	22.7%
**Age**			5001 ∼ 7000	7	12.9%
18 ∼ 27	319	54.9%	7001 ∼ 9000	37	6.4%
28 ∼ 37	128	22.0%	9001 ∼ 11000	39	6.7%
38 ∼ 47	87	15.0%	11001 ∼ 13000	24	4.1%
48 or over	47	8.1%	13001 or over	23	4.0%
**Education**			**Annual travel frequency**		
Junior high school and below	22	3.8%	1–2 times	440	75.7%
Senior high school/college	136	23.4%	3–4 times	115	19.8%
Undergraduate	362	62.3%	5 times and over	26	4.5%
Master degree and above	61	10.5%			

### Variables and measurements

In the process of the questionnaire design, this research ensured the authority and applicability of the questionnaire through the following steps. First, we adopted mature scales verified by international authoritative journals to measure the seven variables in our study. Seven-point Likert-type scales were selected, in which “1” represented “strongly disagree” and “7” represented “strongly agree.” Second, the initial English items were translated into Chinese and modified to satisfy the festival tourism context. Translation-back translation procedures were adopted to ensure that the original meaning of the items remained unchanged.

The main research variables and sources of measurement items were as follows. (1) Tourists’ subjective well-being was adopted from the scale developed by Kwon and Lee (2020) to test the positive effects of tourism on the cultivation of tourists’ well-being (e.g., “I think I am very happy in the experience of festival tourism”); (2) The event design innovation drew on the scales designed by Fu et al. (2018), with the example that there is original scheduling of events, programs and performances; (3) Cultural innovation (e.g., “it innovates the presentation of local cultural elements”) and aesthetic innovation (e.g., “the festival scene is presented with an aesthetic sense”) were all adapted from the scale designed by Hu (2010); (4) The items of experience quality referred to the scale used by Domínguez-Quintero et al. (2020) to measure the experience quality of cultural heritage tourists (e.g., “participating in festival tourism allows me to escape my daily work and do some truly new things”); (5) Perceived festival value was measured from the study of Lin and Lee (2020) (e.g., “This festival tourism is worth spending money, time and energy on”); and (6) Festival authenticity was measured using the scale originally developed by Akhoondnejad (2016) to test the authenticity of traditional festivals for tourists (e.g., “it embodies the traditional culture and art form of festival tourism”).

In Table 2, Cronbach’s α coefficients of all variables were above 0.873 (>0.8), indicating that the reliability of each variable was reliable. Additionally, the load coefficients of the standardized factors in the model were all greater than 0.671 (>0.6). The combined reliability (CR) and average variance extraction (AVE) of latent variables were tested to examine convergent validity and discriminative validity (Fornell and Larcker, 1981). The CR values of all latent variables were above 0.874 (>0.8), and the AVE values were greater than 0.637 (>0.5). Moreover, the results in Table 3 showed that the square root of the AVE value of each variable displayed was greater than the correlation coefficient between this variable and the other variables, confirming that the data had good discrimination validity.

**TABLE 2:** Descriptive statistics and confirmatory factor analysis.

Constructs	Items	Mean	*S.D.*	Standardized factor loading	Standard errors	*t*-value	Cronbach’s α	C.R.	AVE
Event design innovation	EDI1	5.350	1.103	0.789			0.919	0.920	0.657
	EDI2	5.330	1.178	0.815	0.050	21.909***			
	EDI3	5.030	1.121	0.820	0.048	22.107***			
	EDI4	5.140	1.158	0.823	0.049	22.195***			
	EDI5	5.110	1.166	0.819	0.050	22.040***			
	EDI6	5.130	1.146	0.794	0.049	21.185***			
Cultural innovation	CI1	5.380	1.116	0.792			0.910	0.910	0.669
	CI2	5.320	1.071	0.811	0.045	21.943***			
	CI3	5.260	1.107	0.848	0.046	23.292***			
	CI4	5.270	1.095	0.798	0.046	21.466***			
	CI5	5.440	1.037	0.840	0.043	22.983***			
Aesthetic innovation	AI1	5.360	1.076	0.837			0.878	0.881	0.649
	AI2	5.120	1.084	0.829	0.042	23.884***			
	AI3	5.320	1.039	0.812	0.040	23.162***			
	AI4	5.030	1.152	0.739	0.047	20.215***			
Experience quality	EQ1	5.450	1.024	0.847			0.894	0.897	0.637
	EQ2	5.400	1.081	0.843	0.041	25.536***			
	EQ3	5.420	1.117	0.822	0.043	24.462***			
	EQ4	5.310	1.131	0.778	0.045	22.440***			
	EQ5	5.370	1.094	0.689	0.046	18.822***			
Perceived festival value	PFV1	5.350	1.036	0.848			0.873	0.874	0.698
	PFV2	5.280	1.065	0.867	0.041	25.642***			
	PFV3	5.230	1.052	0.789	0.042	22.316***			
Subjective well-being	SWB1	5.550	0.973	0.860			0.935	0.935	0.707
	SWB2	5.450	1.019	0.849	0.038	26.914***			
	SWB3	5.480	0.992	0.833	0.038	25.988***			
	SWB4	5.570	1.034	0.861	0.039	27.621***			
	SWB5	5.480	1.009	0.834	0.039	26.044***			
	SWB6	5.480	1.043	0.808	0.041	24.640***			
Festival authenticity	FA1	5.220	1.186	0.671			0.882	0.885	0.660
	FA2	5.370	1.138	0.799	0.066	17.029***			
	FA3	5.450	1.173	0.888	0.069	18.504***			
	FA4	5.540	1.136	0.873	0.066	18.277***			

***P < 0.001.

**TABLE 3:** Means, standard deviations, correlations and discriminant validity.

Variables	Mean	*S.D.*	1	2	3	4	5	6	7	VIF
(1) Event design innovation	5.239	1.005	**0.811**							
(2) Cultural innovation	5.411	0.979	0.739	**0.818**						2.640
(3) Aesthetic innovation	5.196	0.995	0.722	0.701	**0.806**					2.560
(4) Experience quality	5.411	0.943	0.597	0.628	0.609	**0.798**				2.540
(5) Perceived festival value	5.294	0.955	0.615	0.632	0.593	0.690	**0.835**			2.450
(6) Subjective well-being	5.517	0.920	0.629	0.630	0.613	0.702	0.682	**0.841**		2.270
(7) Festival authenticity	5.395	0.996	0.587	0.629	0.516	0.525	0.531	0.564	0.812	1.630

The significance test of the correlation coefficient all satisfied *p* < 0.001; the bold values were square root of average variance extraction and were located at the diagonal corner of the table.

### Confirmatory factor analyses

To test the structural validity of this model, we used AMOS 23.0 software to analyze the factors of the independent variables (event design innovation, cultural innovation, and aesthetic innovation), mediating variables (experience quality and perceived festival value), dependent variable (subjective well-being), and moderating variable (festival authenticity). The fitness of the seven-factor model hypothesized demonstrated acceptance (χ^2^ = 1159.868, *P* < 0.001; χ^2^/df = 2.447; CFI = 0.957; GFI = 0.889; IFI = 0.957; NFI = 0.929; AGFI = 0.868; RMSEA = 0.050), which laid a good foundation for the next step of data analysis.

### Common method variance

Although common method variance (CMV) was a common systematic error (Richardson et al., 2009), the following measures were taken in the process of collecting data to better control CMV. (1) To obtain a high-quality questionnaire, every variable was adopted from mature scales in authoritative literature, and a number of experts repeatedly checked the items before the survey was finalized; (2) The respondents were clearly informed that the questionnaire was anonymous, and that there were no right or wrong answers; they could answer genuinely; (3) This study introduced unmeasured potential method construction techniques to test whether the data had CMV problems (Richardson et al., 2009). First, the research tested the various indicators of the original 7-factor model fit and then included the method factors that were not related to the 7 factors so that all test items had a load on the method factors. After including the method factors, the results showed that the fitting indices of this model (χ^2^ = 932.045, *P* < 0.001; χ^2^/df = 2.114; CFI = 0.969; GFI = 0.912; IFI = 0.969; NFI = 0.943; AGFI = 0.888; RMSEA = 0.044) were better than those of the original 7-factor model. In addition, the adjusted chi-square value is lower than the critical value of 0.05. Therefore, there were no serious CMV problems in this study.

## Results

The statistics of the mean, standard deviation, and correlation coefficient of all variables in this study were shown in Table 3. There was a significant and positive correlation between the 7 variables. However, the correlation coefficient between individual structures was large, such as the relationship between event design innovation and cultural innovation (*r* = 0.739, *p* < 0.001). Therefore, the variance inflation factor (VIF) was used to examine collinearity. Table 3 showed that no VIF values exceeded 2.64 (<5.0), indicating that there was no collinearity problem in this study (Podsakoff et al., 2003).

**FIGURE 2:**
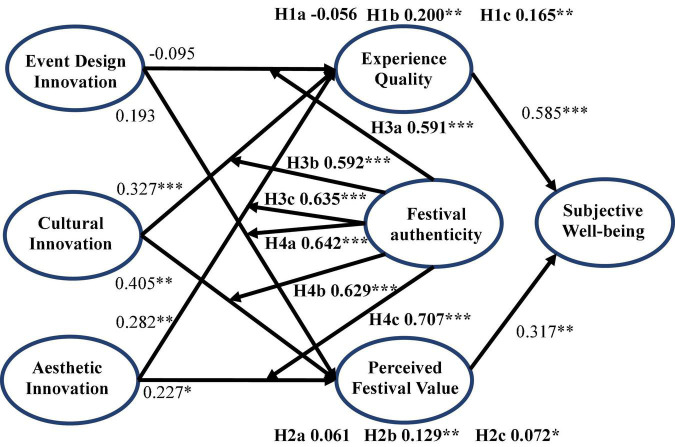
Research model results. *P < 0.05, **P < 0.01, ***P < 0.001.

Structural equation modeling (SEM) was introduced to examine the hypothetical model because it could more comprehensively test complex models and avoid some deviations (Liu, 2018). Therefore, AMOS 23.0 software was used to test the path effects and standard error (SE). Moreover, we conducted a bootstrap approach with 2,000 resampling to obtain a 95% confidence interval (CI) to test the mediation effects and moderation effects. In addition, the overall model of this study showed a good fit (χ^2^ = 940.354, *P* < 0.001; χ^2^/df = 2.576; CFI = 0.959; GFI = 0.898; NFI = 0.935; AGFI = 0.878; RMSEA = 0.052). The specific hypothesis test results were presented in Figure 2.

As illustrated in Table 4 and Figure 2, the direct effect of event design innovation on experience quality was not significant (β = −0.095, *P* = 0.336), and neither was there a significant direct effect of event design innovation on festival value (β = 0.193, *P* = 0.089). In addition, the two variables of experience quality and perceived festival value had no significant mediating effects between event design innovation and subjective well-being (β = −0.056, *P* = 0.334; β = 0.061, *P* = 0.067). Therefore, H1a and H2a were unsupported.

**TABLE 4:** Mediation effect test.

Hypothesis path	Estimates	Standard error	Bias-corrected 95% CI		Percentile 95% CI		Results
			Lower	Upper	Lower	Upper	
H1a: EDI → EQ → SWB	–0.056	0.060	–0.176	0.528	–0.180	0.049	Unsupported
H1b: CI → EQ → SWB	0.200**	0.080	0.052	0.366	0.041	0.351	Support
H1c: AI → EQ → SWB	0.165**	0.070	0.052	0.338	0.045	0.322	Support
H2a: EDI → PFV → SWB	0.061	0.041	–0.004	0.162	–0.007	0.159	Unsupported
H2b: CI → PFV → SWB	0.129**	0.054	0.043	0.267	0.032	0.245	Support
H2c: AI → PFV → SWB	0.072*	0.041	0.011	0.173	0.010	0.171	Support

*P < 0.05, **P < 0.01. EDI, event design innovation; CI, cultural innovation; AI, aesthetic innovation; EQ, experience quality; PFV, perceived festival value; SWB, subjective well-being.

There was a significant positive correlation between cultural innovation and experience quality (β = 0.327, *P* < 0.001) and between experience quality and subjective well-being (β = 0.585, *P* < 0.001). Perceived festival value and cultural innovation were significantly positively correlated (β = 0.405, *P* < 0.01), and it was also significantly and positively correlated with subjective well-being (β = 0.317, *P* < 0.01). Therefore, H1b and H2b were supported.

Aesthetic innovation had a significant positive impact on experience quality and perceived festival value (β = 0.282, *P* < 0.01; β = 0.227, *P* < 0.05). In this path from aesthetic innovation to subjective well-being, after adding the mediating variables (i.e., experience quality and perceived festival value), the 95% confidence interval of the mediating effect between aesthetic innovation and subjective well-being obviously did not include 0. Therefore, H1c and H2c were supported.

**FIGURE 3:**
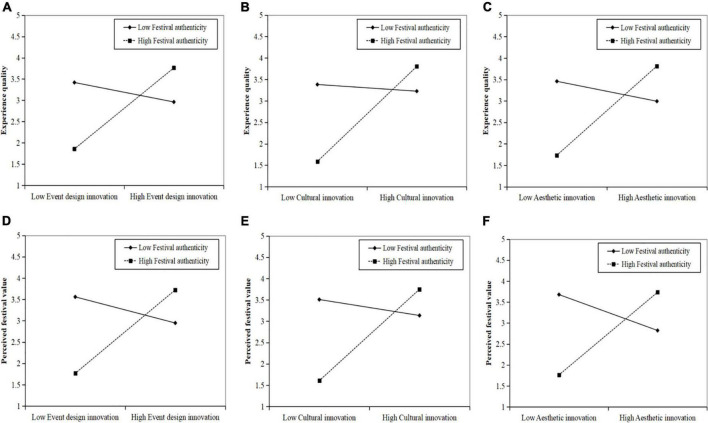
**(A)** Interaction of event design innovation and festival authenticity on experience quality. **(B)** Interaction of cultural innovation and festival authenticity on experience quality. **(C)** Interaction of aesthetic innovation and festival authenticity on experience quality. **(D)** Interaction of event design innovation and festival authenticity on perceived festival value. **(E)** Interaction of cultural innovation and festival authenticity on perceived festival value. **(F)** Interaction of aesthetic innovation and festival authenticity on perceived festival value.

Table 5 and Figure 3 showed the moderating effects of festival authenticity. The result indicated that the interaction items between independent variables and festival authenticity (i.e., event design innovation × festival authenticity, cultural innovation × festival authenticity, and aesthetic innovation × festival authenticity) had significant positive impacts on experience quality (β = 0.591, *P* < 0.001; β = 0.592, *P* < 0.001; β = 0.635, *P* < 0.001), which confirmed the moderating roles of festival authenticity. Therefore, H3a, H3b, and H3c were supported. Moreover, consistent with the hypotheses, the interaction terms (i.e., event design innovation × festival authenticity, cultural innovation × festival authenticity, and aesthetic innovation × festival authenticity) had significant positive impacts on the perceived festival value (β = 0.642, *P* < 0.001; β = 0.629, *P* < 0.001; β = 0.707, *P* < 0.001), which indicated the moderating role of festival authenticity. Therefore, H3b–H4c were all supported. Finally, some simple slope figures demonstrated the specific moderating roles of authenticity.

**TABLE 5:** The moderating effects of festival authenticity.

Specific path		Estimates	Standard error	Results
H3a	EDI → EQ	0.362***	0.077	Support
	FA → EQ	−0.190**	0.078	
	EDI × FA → EQ	0.591***	0.008	
H3b	CI → EQ	0.514***	0.070	Support
	FA → EQ	−0.304***	0.074	
	CI × FA → EQ	0.592***	0.007	
H3c	AI → EQ	0.403***	0.056	Support
	FA → EQ	−0.225**	0.063	
	AI × FA → EQ	0.635***	0.007	
H4a	EDI → PFV	0.336***	0.079	Support
	FA → PFV	−0.254**	0.079	
	EDI × FA → PFV	0.642***	0.008	
H4b	CI → PFV	0.442***	0.073	Support
	FA → PFV	−0.322***	0.078	
	CI × FA → PFV	0.629***	0.008	
H4c	AI → PFV	0.279***	0.060	Support
	FA → PFV	−0.251**	0.068	
	AI × FA → PFV	0.707***	0.008	

**P < 0.01, ***P < 0.001. EDI, event design innovation; CI, cultural innovation; AI, aesthetic innovation; EQ, experience quality; PFV, perceived festival value; SWB, subjective well-being; FA, festival authenticity.

## Conclusion and discussion

Based on Arnold’ s theory of emotion, this paper uses the structural equation model to test the theoretical model of the formation of tourists’ subjective well-being in response to festival tourism innovation. Our research has produced the following fruitful findings:

### Conclusion

First, festival tourism innovation can enhance tourists’ subjective well-being by improving the experience quality and the perceived festival value. Innovation is an effective way to stimulate tourists’ curiosity and interest (Zhang et al., 2019a), which can increase the added value of products and fulfill tourists’ new experience needs. This study proves that experience quality and perceived festival value are the cognition and evaluation of festival tourists after being stimulated by innovation, and they are the critical link in the formation of tourists’ subjective well-being. On the one hand, with the rapid development of cultural and creative industries, festival cultural innovation has received a great response at the consumer level, creating a huge experience space for festival tourists (Zhang et al., 2019b,Zhang et al., 2021). At the same time, the various sensory enjoyments caused by aesthetic innovation can positively affect the perception evaluation of tourists, and the aesthetic stimulation it brings has a positive impact on the perceived value of tourists (Aşan et al., 2020). On the other hand, the experience quality and perceived festival value have a certain superimposing effect, which can affect the positive emotions of tourists, forming tourists’ subjective well-being. Therefore, festival tourism innovation can improve the experience quality and perceived festival value and ultimately increase tourists’ subjective well-being.

Second, in the relationship between event design innovation and tourists’ subjective well-being, neither experience quality nor the perceived festival value play a mediating role, but they can contribute a positive influence in the context of festival authenticity. Although innovation in event design can provide tourists with more space for participation and interaction (Hjalager, 2015; Romão and Nijkamp, 2019), it is difficult to improve the experience quality and perceived value of tourists by relying only on event design innovation in the context of festival tourism destination. The reason may be that the effect of event design innovation on festival tourists is not obvious enough. Especially, when tourists have obtained entertainment experience in their daily life, they are easy to associate and compare the activity experience during the festival tourism with the previous ones. As a result, there are higher expectations and requirements for festival activities, which leads to a fact that the event design innovation of festival tourism is not enough to meet the authentic festival experience needs of tourists. However, this study finds that under the moderating effect of festival authenticity, event design innovation is a positive antecedent factor. The reason is that festival activities with authenticity guarantees do not deviate from local characteristics and cultural meanings but rather meet tourists’ local cultural experience needs and desire to have an original experience (Zhang et al., 2019b). Among the benefits of festival authenticity, tourism innovation presents characteristic festival performances and festival content and enhances the experience quality and festival value. In summary, the findings further support the argument that “authenticity is a key factor in determining the success of innovation under certain circumstances” in festival tourism (Keiningham et al., 2019).

Third, festival authenticity has played a significant moderating role in the influence of festival tourism innovation on tourists’ experience quality and perceived festival value. The stronger the festival authenticity is, the more authentic the festival culture, art, and atmosphere felt by tourists (Brida et al., 2013), which improves their acceptance of festival tourism innovation and experience quality. During the experience, tourists not only increase their enthusiasm and interest in participation but also take the initiative to further understand the image of festival tourism destinations, cultural uniqueness and experience contents. Furthermore, the more authentic the festival, the more tourists realize that innovation is not a cultural copy or a commercial intrusion. It strengthens tourists’ recognition and trust in festival innovations, induces their positive emotions and improves their evaluation of tourism benefits (Brida et al., 2013; Akhoondnejad, 2016). Thus, festival authenticity can strengthen the positive impact of festival tourism innovation on perceived value.

### Theoretical contribution

First, this paper fills research gaps on the deep-level emotional response of festival tourists, and expands the theoretical system of tourists’ positive emotion research in the process of festival tourism from the perspective of tourism innovation. Existing literature focuses on the importance of tourist emotional responses to sustainable tourism development (Kruger and Viljoen, 2021). Different from tourists’ satisfaction, loyalty (Tanford and Jung, 2017) or revisit intention (Kruger and Viljoen, 2021), which are generally concerned at present, this paper explores the deep emotional response of festival tourists: subjective well-being. It further fills research gap on the emotional response of festival tourists (Laing, 2018), and provides new ideas for the sustainable development of festival tourism. In addition, when exploring the influencing factors of tourists’ positive emotional response, scholars majorly discuss the influence of festival tourism culture and environment. They are less likely to consider innovation as a critical stimulus (Hu, 2010). This study analyses the logic of festival tourism innovation on the positive emotions of tourists and clarifies the key role of cultural innovation and aesthetic innovation. This coincides with the scholars’ viewpoints that festival culture and aesthetics should be valued (Trinh and Ryan, 2016).

Second, this study clarifies the internal mechanism from “festival tourism innovation” to “tourists’ subjective well-being,” highlights the dual mediating roles of experience quality and perceived festival value, and expands the application and boundaries of Arnold’ s theory of emotion. Research shows that experience quality and perceived festival value are key evaluation components before the formation of tourists’ subjective well-being. Cole and Scott (2004) believe that the tourism experience has a strong cumulative effect. This study reveals that experience quality can enhance tourists’ subjective well-being. Moreover, Schwartz and Sortheix (2018) propose that there is a contextual relationship between tourists’ pursuit of value and life satisfaction, calling on scholars to pay attention to tourists’ perception of value and subjective well-being. Therefore, this study affirms and deepens the current research conclusions and responds to the research deficiencies and prospects raised in the literature. It improves the research on the inducing factors of tourists’ subjective well-being in the context of festival tourism, clarifies the influential process mechanism of festival tourism innovation on tourists’ subjective well-being, and expands the fields of application and boundaries of Arnold’ s theory of emotion.

Third, it reveals the contextual role and influence boundary of authenticity in the relationship between festival tourism innovation, experience quality and perceived festival value and provides new ideas for the collaborative advancement of festival tourism’s “innovative development and authentic inheritance.” As a significant research topic in the tourism field, authenticity usually explains the formation logic of tourists’ positive behaviors. Previous studies have proven that authenticity can stimulate tourists’ motivation, influence tourists’ emotions and trigger tourists’ trust and loyalty (Akhoondnejad, 2016; Stepchenkova and Belyaeva, 2021). However, this study explores the contextual role of authenticity in festival tourism and proves the effect of authenticity on tourist experience in different tourism subdivisions. It further enriches the research content of tourism authenticity. Furthermore, Keiningham et al. (2019) argue that the relationship between innovation and authenticity has not been reasonably explained. However, this study finds that in festival tourism, only when festival authenticity is guaranteed can event design innovation lead to tourists’ positive perceptions and evaluations. It further clarifies the logical relationship between “innovative development and authentic inheritance.”

### Managerial implications

First, to enhance tourists’ subjective well-being, festival tourism destinations should be aware of the important role of cultural and aesthetic innovations in festival tourism. Destinations should strengthen the management and marketing of festival tourism cultural innovation, actively build or introduce creative talents, teams, and products (Zhang et al., 2019a). It is necessary to use social media platforms to upload animations, pictures and short videos of festival celebrations or development history, which will show the innovative elements of festival culture (Zhang et al., 2019a). Moreover, destination managers should pay attention to the aesthetic innovation and actively display aesthetic festival elements to strengthen the multi-dimensional sensory experience of tourists (Zhang and Xu, 2020). Examples include combining artificial intelligence or virtual reality; offering festival music, dance or other content. In addition, we advocate that tourist destinations strengthen the aesthetic design and innovation of festival features, such as buildings, public facilities, food, souvenirs and transportation, to enhance the image and brand perception of the destination for festival visitors (Aşan et al., 2020).

Second, it is feasible for festival tourism destinations to strengthen the catalytic effect of festival authenticity and handle the relationship between festival tourism innovation and authentic inheritance. Destination managers should coordinate the deep integration of culture with festival products, services and markets. This is a good way to place cultural authenticity at the core, guiding visitors to capture real festival clues and choose products that can awaken their festival impressions while avoiding excessive commercialization. We also advocate that tourist destinations actively embed the festival’s culture, imagery and history into cultural and creative products and services (Girish and Chen, 2017; Zhang et al., 2019c). In addition, we suggest that destination managers present festival history, culture, folk customs and local characteristics (such as special cuisine and landscapes) in innovative and creative forms to strengthen value perception and local identity and improve festival visitors’ understanding and awareness of local culture.

Third, destinations need to improve tourists’ experience quality and their perception of festival value to ensure that their subjective well-being is enhanced. It is necessary for tourist destination organizers to ensure the quality and value of the tourism experience through product innovation, cultural creativity and atmosphere creation. For example, destination organizers can combine historical festival stories and local tourism resources to create destination-specific products and optimize tourists’ cultural experience. Managers need to design festival themes and create a good atmosphere to provide tourists with a comfortable travel environment, special festival tourism products and entertainment services that are conducive to expanding tourists’ perception of the festival’s value (Zhang et al., 2019c).

### Limitations and future research suggestions

This research has revealed the mechanism of festival tourism innovation on tourists’ subjective well-being and enriched the theoretical system of festival tourism research, but some limitations should be recognized and solved in future studies. First, this research mainly discusses the effect of tourism innovation on tourists’ subjective well-being in the field of festival tourism, but whether the theoretical model and influence path are relevant in other tourism areas remain to be further tested. Second, this paper selects event design innovation, cultural innovation and aesthetic innovation to represent festival tourism innovation and explores the influence path of innovation on tourists’ subjective well-being. The effect of more innovative elements can be compared in depth in the future. Furthermore, tourists’ subjective well-being is a deep-seated emotion that is affected by various factors, such as personal wishes and the environment (Anglim et al., 2020). Future research can conduct situational experiments or use fuzzy-set qualitative comparative analysis (fsQCA) to analyze how the element configuration affects festival tourists’ subjective well-being, further excavating the inner logic of “innovative development and authentic inheritance.”

## Data availability statement

The raw data supporting the conclusions of this article will be made available by the authors, without undue reservation, to any qualified researcher.

## Author contributions

S-NZ: original idea, writing—reviewing and editing, conceptualization, methodology, and data collection. FD: writing—original draft preparation, literature review, data curation, and software. Both authors contributed to the article and approved the submitted version.
